# Profibrotic Effects of Endothelin-1 on Fibroblasts Are Mediated by Aldosterone In Vitro: Relevance to the Pathogenesis and Therapy of Systemic Sclerosis and Pulmonary Arterial Hypertension

**DOI:** 10.3390/biomedicines10112765

**Published:** 2022-10-31

**Authors:** Giuseppe Argentino, Alessandro Barbieri, Ruggero Beri, Caterina Bason, Andrea Ruzzenente, Oliviero Olivieri, Elisa Tinazzi, Antonio Puccetti, Claudio Vitali, Nicoletta Del Papa, Simonetta Friso, Claudio Lunardi

**Affiliations:** 1 Department of Medicine, University of Verona, 37134 Verona, Italy; 2 Department of Laboratory Medicine, Boston Children’s Hospital, Harvard Medical School, Boston, MA 02115, USA; 3 Department of Biomedical and Surgical Sciences, University of Verona, 37134 Verona, Italy; 4 Department of Experimental Medicine, Section of Histology, University of Genova, 16132 Genova, Italy; 5 Rheumatology Outpatients Clinics, Humanitas ‘Mater Domini’ Hospital, 21053 Castellanza, Italy; 6 Scleroderma Clinic, ASST Gaetano Pini–CTO, 20122 Milano, Italy

**Keywords:** endothelin-1, aldosterone, reactive oxygen species, systemic sclerosis, pulmonary arterial hypertension, fibroblasts

## Abstract

Endothelin-1 (ET-1) is a vasoactive and profibrotic peptide that plays a pivotal role in diseases such as systemic sclerosis (SSc) and pulmonary arterial hypertension (PAH), by inducing fibrosis and vascular remodeling. Such effects may be sustained by the induction of aldosterone production and reactive oxygen species (ROS). We have used fibroblasts obtained from skin of healthy donors and SSc patients and commercial fibroblasts from lung to evaluate whether ET-1 is able to stimulate ROS production directly or indirectly through aldosterone induction. We found that ET-1 receptors are present in all types of fibroblasts analyzed, whereas the expression of mineralocorticoid receptor (MCR) is lower in dermal fibroblasts from healthy donors (HDFs) compared to fibroblasts derived from lung (HPFs) or from skin of SSc patients (SScHDFs). ET-1 induces ROS production in HDFs and SScHDFs after 24 h of incubation involving its receptor B (ETB), whereas aldosterone exerts its effects after 40 min of incubation. Moreover, ROS production was inhibited by the pre-incubation of cells with MCR inhibitor. Our results indicate that ET-1 induces ROS indirectly through aldosterone production suggesting that aldosterone may play a pivotal role in the pathogenesis of SSc and PAH.

## 1. Introduction

Endothelin-1 (ET-1) is a peptide consisting of 21 amino acids; its C-terminus is hydrophobic, and the N-terminus contains two cysteine bridges [1]. It is predominantly a vasoconstriction mediator, but it is also involved in inflammation, cell adhesion, fibrosis, and angiogenesis [2]. Four isoforms of endothelin have been identified: ET-1 (produced by a large number of different cells as smooth muscle cells, fibroblasts, myofibroblasts, mastocytes, monocytes/macrophages, polymorphonucleate cells, dendritic cells, and others), ET-2 (produced by intestinal and renal cells), ET-3 (found in central nervous system), and ET-4 (little is known in the literature) [1,3]. It has been shown that ET-1 is mainly involved in fibrosing diseases such as systemic sclerosis (SSc); carcinogenesis; and cardiovascular, renal and pulmonary pathologies (including pulmonary hypertension) [1,4,5,6]. ETA, ETB, and ETC are G-protein-coupled receptors that mediate the endothelin effects. ETA and ETB bind ET-1, ET-2, and ET-3, but ETA is more specific for ET-1, while ETB binds the three ET isoforms with the same specificity. Little is known about the binding specificity of ETC. We have already reported their presence on immune cells [6]. The various effects of this peptide, including the modulation of inflammation, are regulated by the bound receptor, the stimulated cell, and the tissue conditions [1,2,6,7]. Endothelial cells (ECs) and smooth muscle cells (SMCs) of vasal tone express differently ET receptors: ETB is exclusive on ECs, while, on SMCs, both ETA and ETB are present but ETB is predominant. The balance of ET-1 actions in vasal tissue is important in order to maintain vascular tone: ETA on SMCs mediate contraction, promoting the production of inositol 1,4,5-trisphosphate (IP3), and this effect is balanced by ETB on ECs through the production of nitric oxide (NO) [1]. All of the causes that alter this balance lead to vasoconstriction. ET-1 is able to stimulate the production of collagen, the expression of intracellular adhesion molecule 1 (ICAM-1), and the trans-differentiation of fibroblasts into myofibroblasts [8,9] It is known that the selective blocking of ETA or ETB does not inhibit the collagen production mediated by ET-1, while combined ETA/ETB antagonism reverts the fibrotic fibroblast phenotype to a normal phenotype. This would suggest the presence of cross-talk between the two receptors [8,10]. The involvement of ET-1 in the development of fibrotic and vascular events has been hypothesized because SSc and/or PAH patients show an increased concentration of ET-1 in the sera [11,12,13]. Indeed, ETA/ETB antagonists are currently used in the treatment of SSc skin ulcers and of PAH [14,15]. In healing tissues, activated fibroblasts acquire a contractile phenotype due to an increased expression of α-smooth muscle actin (α-SMA), and for this reason, they were called myofibroblasts [16]. ET-1 seems to be involved in the endothelial-to-mesenchymal transition (EndoMT) that is an important aspect of vasculopathy and lung fibrosis. Indeed, ECs change their phenotype into a mesenchymal and myofibroblastic phenotype; ECs dissociate from the monolayer of tightly cohesive cells at the lumen of the vessels and migrate toward the inner wall. During this migration, ECs lose specific endothelial markers (CD31, CD34, and VE-cadherin) and express mesenchymal or myofibroblastic markers like α-SMA and vimentin [17,18,19]. Patients with primary hyperaldosteronism show cardiac remodeling due to the transformation of myocardial tissue into fibrotic tissue, mainly triggering the process of the transition of fibroblasts and endothelial cells into myofibroblasts. This event leads to an increased production of collagen and extracellular matrix proteins. Furthermore the stimulation of monocytes/macrophages with the subsequent activation of inflammation, necrosis, and the genesis of reparative fibrous tissue has been shown to contribute to the generation of fibrotic tissue [20,21,22,23].

The aldosterone production in ECs due to high levels of ET-1 that eventually stimulates the production of reactive oxygen species (ROS) is an interesting aspect to be investigated. Nowadays, aldosterone is considered a mediator of the cardiopulmonary vascular phenotype in PAH; indeed, this steroid hormone has been detected in high levels in experimental models and PAH patients. This is not only due to the increased adrenal production of aldosterone but also of extra-adrenal synthesis found in the pulmonary vessels [2,24,25]. ET-1, which is upregulated in PAH, is known to induce the production of aldosterone; indeed hyperaldosteronism is present in PAH [26]. These persistently elevated aldosterone levels activate the mineralocorticoid receptor (MCR), contributing to vascular remodeling, altered vascular reactivity, and cardiac dysfunction. In the PAH rat monocrotaline model, ET-1 levels in plasma and lungs are increased; this is associated with high aldosterone levels in the same fluid/tissue. ET-1 is able to stimulate extra-adrenal pulmonary aldosterone synthesis [2]. Pulmonary endothelial ROS have been implicated in PAH and have been shown to disrupt NO-dependent vasodilatory signaling pathways to promote pulmonary vessel vasoconstriction, the muscularization of pulmonary arterioles, and perivascular fibrosis [27,28]. In pulmonary tissue, the ETB-mediated eNOS activation results in the production of endogenous NO [29]; however, how ROS induces the decreased production of NO in PAH is not clear. Near the C-terminus, ETB contains Cys405 in its intracellular domain; this amino acid is a cysteinyl thiol with a pivotal role in ETB signal transduction [30,31]. In a model proposed by Maron et al. [2], the Cys405 oxidative reduction due to aldosterone-induced ROS is responsible for the failure of ETB–eNOS activation in the pulmonary artery endothelial. The oxidative reduction of ETB Cys405 due to ROS blocks the interaction between ETB and eNOS; this oxidative modification of Cys405 in pulmonary artery ECs disables ETB–eNOS activation, promoting pulmonary vascular dysfunction; this highlights the importance of Cys405 for the activation of eNOS [2]. The renin–angiotensin–aldosterone system (RAAS) has not been extensively investigated in SSc patients, and there are conflicting data on the angiotensin II levels in SSc patients. Regarding fibroblasts, the data show an increased expression of angiotensinogen in SSc fibroblasts compared to healthy controls [32]. There is clear evidence that the RAAS is activated in PAH. In preclinical studies, a rat model with PH showed the increased expression and activity of angiotensin-converting enzyme (ACE) in pulmonary vessels [33].

The aim of this work was to clarify the possible role of ET-1 in the pathogenesis of SSc and PAH and to investigate the effects of this peptide on lung and skin fibroblasts. Often ET-1 action is analyzed on tunica intima and media (ECs and SMCs, respectively), ignoring the possible implication of tunica adventitia. For this purpose, we analyzed the activation of fibroblasts through the production of the extracellular-matrix protein since fibrosis is the main characteristic of scleroderma. Moreover, we investigated the possible involvement of ET-1 on ROS production, focusing our attention on the link between ET-1 and aldosterone and their increased levels in SSc and PAH. To focus on the districts most affected by fibrosis in SSc (skin) and PAH (lungs), we decided to investigate fibroblasts. Where possible, cells from biopsies were used.

## 2. Materials and Methods

### 2.1. Cell Cultures and Treatments

This study was performed using human pulmonary fibroblasts (HPFs; PromoCell, Heidelberg, Germany), dermal fibroblasts obtained by skin biopsy from two healthy donors (human dermal fibroblasts, HDFs) and from four SSc patients affected by both limited or diffuse cutaneous form of the disease (lSScHDFs and dSScHDFs respectively). Cells were grown to confluence at 37 °C and 5% CO_2_ by using fibroblast growth medium 2 (PromoCell, Heidelberg, Germany) for the commercial line while in DMEM (ThermoFischer Scientific/Gibco, Waltham, MA, USA) +10% FBS (Sigma-Aldrich, St. Louis, MO, USA) +1% penicillin streptomycin (pen–strep; ThermoFischer Scientific/Gibco, Waltham, MA, USA) for the cells obtained from skin biopsies. Cells were passaged by using accutase (Millipore, Billerica, MA, USA), and experiments were performed five times with cells from passages 4 to 10.

ET-1 (100 nM equal to 0.25 μg/mL) (Tocris Bioscience, Bristol, UK) and the selective ETA endothelin receptor antagonist BQ123 (1.5 μM equal to 0.92 μg/mL) (Sigma-Aldrich, St. Louis, MO, USA) were suspended in RPMI (Lonza, Basel, Switzerland); the selective ETB endothelin receptor antagonist BQ788 (1.5 μM equal to 0.99 μg/mL) (Sigma-Aldrich, St. Louis, MO, USA), aldosterone (100 nM equal to 0.04 μg/mL) (Sigma-Aldrich, St. Louis, MO, USA) and the MCR inhibitor spironolactone (10 μM equal to 4.16 μg/mL) (Sigma-Aldrich, St. Louis, MO, USA) were suspended in DMSO (Sigma-Aldrich, St. Louis, MO, USA).

### 2.2. Patients and Healthy Donors

Fibroblasts were isolated from skin biopsies of two healthy donors and of four SSc patients with PAH after written informed consent (Table 1). Healthy donors’ biopsies were collected in the General Surgery Unit, University Hospital of Verona. SSc patients attended the Autoimmune Diseases Unit, University Hospital of Verona. The patients fulfilled the American College of Rheumatology (ACR) and the European League Against Rheumatism (EULAR) classification criteria for SSc [34]; the classification in diffuse and limited cutaneous forms was based on LeRoy’s criteria [35,36].

### 2.3. Fibroblasts Isolation

Biopsy samples (0.6 cm of the forearm skin) were placed immediately in DMEM +1% pen–strep. In a sterile hood, the skin samples were transferred in 1 mL digestion medium (DMEM +20% FBS +0.25% collagenase type I +0.05% DNAse I +1% pen–strep; collagenase type I and DNAse I were purchased from Sigma-Aldrich, St. Louis, MO, USA) then they were placed overnight in 37 °C tissue-culture incubator. Next day, the samples were vortexed for 20 s, and 7 mL of culture medium (DMEM +20% FBS +1% pen–strep) was added in a sterile hood. Then entire contents were transferred in T75 tissue culture flasks (BD Bioscience/Falcon, San Josè, CA, USA) that were placed in a 37 °C tissue-culture incubator for 72 h. On day 6, 7 mL of culture medium (DMEM +10% FBS +1% pen–strep) was added. Subsequently, the cells were subcultured for the first time, and the medium used was DMEM +10% FBS +1% pen–strep.

### 2.4. RNA Isolation and RT-PCR

RT-PCR were performed to evaluate the mRNA presence of ETA and ETB receptors, MCR and α-SMA, at the transcriptional level.

HPFs and HDFs were cultured in 6-well plates and were stimulated by ET-1 for 24 h. Using TRI Reagent (Sigma-Aldrich, St. Louis, MO, USA), the total RNA was obtained and then used for retrotranscription in cDNA. The amplification of cDNA was performed using AmpliTaq Gold PCR MasterMix (Applied Biosystems, Foster City, CA, USA) and GeneAmp PCR System 9700 thermocycler (Applied Biosystems, Foster City, CA, USA). The following primers (Sigma-Aldrich, St. Louis, MO, USA) were used for ETA, ETB, MCR, and α-SMA detection, and Vimentin was used as an internal control.
ETA: forward       5’-ATGCACAACTATTGCCCACA-3’    reverse        5’-GGACAGGATCCAGATGGAGA-3’ ETB: forward       5’-GCACATCGTCATTGACATCC-3’    reverse        5’-CAGAGGGCAAAGACAAGGAC-3’ MCR: forward     5’-AGGCTACCACAGTCTCCCTG-3’     reverse       5’-GACTGGAGATTTTACACTGC-3’ α-SMA: forward   5’-GGAATCCTGTGAAGCAGCTC-3’              reverse     5’-GAAGGAATAGCCACGCTCAG-3’

### 2.5. Western Blot Analysis

TRIS, Tween 20, NaCl, MgCl2, β-mercaptoethanol, glycine, glycerol, bromphenol blue, sodium dodecil sulphate (SDS), 30% acrylamide/bis solution, methanol, and ammonium persulfate (APS) were purchased by Sigma-Aldrich (St. Louis, MO, USA); Temed was purchased from GE Healthcare Life Biosciences (Little Chalfont, UK); Dulbecco’s phosphate buffered saline (DPBS) was from Lonza (Basel, Switzerland).

Western blot analyses were performed to confirm the expression of ETA and ETB receptors on cell surface and to evaluate the presence of MCR and Cytochrome P450 11B2 (CYP11B2—aldosterone synthase) in the intracellular space of HPFs, HDFs and SScHDFs. The total protein lysate was obtained using RIPA buffer +1% protease inhibitors mix (Roche, Penzberg, Germany). Equal amounts of protein samples (20 μg) were separated by electrophoresis with 10% acrylamide gel under reducing conditions and transferred to a nitrocellulose membrane (GE Healthcare Life Biosciences/Amersham Biosciences, Little Chalfont, UK). The membranes were then incubated in blocking buffer containing 5% non-fat dry milk powder (Sigma-Aldrich, St. Louis, MO, USA). ETA, ETB, MCR, and CYP11B2 were detected by incubating the membrane with 1:100 polyclonal primary antibody anti-ETA (Acris Antibodies, GmbH, Herford, Germany) or 1 μg/mL polyclonal anti-ETB (Lifespan Biosciences, Seattle, WA, USA) or 1:500 polyclonal primary antibody anti-MCR (Abcam, Cambridge, UK) or 1:60 polyclonal primary antibody anti-CYP11B2 (Abcam, Cambridge, UK) overnight at 4 °C. Furthermore, 1:100 polyclonal primary antibody anti-β-actin (Sigma-Aldrich, St. Louis, MO, USA) was used as internal control. Membranes were then incubated with a 1:3000 anti-rabbit IgG-HRP for ETA (GE Healthcare, Freiburg, Germany) or a 1:3000 anti-sheep IgG-HRP for ETB (Bethyl Laboratories, Inc. Montgomery, TX, USA) or 1:1000 anti-rabbit IgG-HRP (Santa Cruz Biotechnology, Dallas, TX, USA) for MCR, CYP11B2, and β-actin for 1 h at room temperature. The detection was performed using the ECL detection system (GE Healthcare Life Biosciences/Amersham Biosciences, Little Chalfont, UK) and the Image Quant Las Mini 4000 Digital Imaging System (GE Healthcare Life Biosciences/Amersham Biosciences, Little Chalfont, UK), while densitometric analysis was carried out using the Image Quant TL software (GE Healthcare Life Biosciences/Amersham Biosciences, Little Chalfont, UK).

### 2.6. Flow Cytometry

FACSCanto II cytometer (BD Bioscience/Falcon, San Josè, CA, USA) was used to detect the expression of ETA and ETB receptors and to evaluate the oxidative stress induced by ET-1 or aldosterone. The data were analyzed by FlowJo software (Treestor, Ashland, OR, USA).

HPFs, HDFs, and SScHDFs were incubated with the same anti-ETA or anti-ETB polyclonal primary antibodies used for Western blot analyses and stained with R-Phycoerythrin (PE) secondary monoclonal anti-rabbit or anti-sheep IgG (R&D Systems, Minneapolis, MN, USA) respectively; as negative controls were used, cells were incubated only with secondary antibodies.

The detection of intracellular oxidative stress formation was performed using 5-(and-6)-chloromethyl-2’,7’-dichlorodihydrofluorescein diacetate (CM-H2DCFDA; Invitrogen, Oregon, USA), 50 μM dissolved in DMSO. The ROS production in HPFs, HDFs, and SScHDFs grown with 10% human serum (Invitrogen, Oregon, USA) was measured after 40 min (short) or 24 h (long) ET-1 stimulation and after incubation with CM-H2DCFDA for 30 min. The individual receptor block was evaluated by pre-incubation with BQ123 (anti ETA) and/or BQ788 (anti ETB) for 30 min. ROS production was also evaluated after 40 min stimulation with aldosterone (ALDO) with or without 30 min of spironolactone (SPIRO) pre-incubation. Moreover, cells underwent long ET-1 stimulus together with spironolactone. H2O2 was used as an internal positive control in our experiments, and cells were washed with Hank’s balanced salt solution (HBSS; ThermoFischer Scientific/Gibco, Waltham, MA, USA).

### 2.7. Enzyme-Linked Immunosorbent Assay

Collagen-1, TGF-β, and PDGF were measured in HPFs and HDFs supernatants by an enzyme-linked immunosorbent assay (ELISA) before and after short or long stimulation with ET-1. Collagen-1 ELISA kit was purchased from BlueGene Biotech (Shanghai, China), while TGF-β and PDGF ELISA kits were purchased from R&D Systems (Minneapolis, MN, USA). The optical densities of enzymatic reaction were determined with a TECAN Sunrise III (Tecan, Männedorf, CH) plate reader.

Aldosterone was measured in HPFs, HDFs, and SScHDFs supernatants by competitive ELISA (R&D Systems, Minneapolis, MN, USA) before and after 40 min (short) or 24 h (long) stimulation with ET-1 with or without ETA and/or ETB receptor blockers.

### 2.8. Protein Carbonyl Content Assay

The detection of intracellular ROS formation was evaluated indirectly by measuring carbonyl groups resulting from the oxidation of proteins by oxidative injury using a protein carbonyl content assay (Sigma-Aldrich, St. Louis, MO, USA). HDFs and SScHDFs were stimulated with ET-1 with or without ETA and/or ETB receptor blockers (pre-incubation of 30 min) or aldosterone with or without spironolactone (pre-incubation of 30 min) before and after 40 min (short) incubation but also after 24 h (long) stimulation with ET-1 with or without ETA and/or ETB receptor blockers or spironolactone (pre-incubation of 30 min).

### 2.9. Statistical Analysis

GraphPad Prism software (GraphPad Software Inc., La Jolla, CA, USA) was used to perform all statistical analysis. Data with a normal distribution were analyzed with Student’s t-test and were expressed as a mean ± standard deviation. No more than 2 groups were compared simultaneously. A difference between groups with a *p* < 0.05 was considered statistically significant.

## 3. Results

### 3.1. ET-1 Receptors

**Figure 1:**
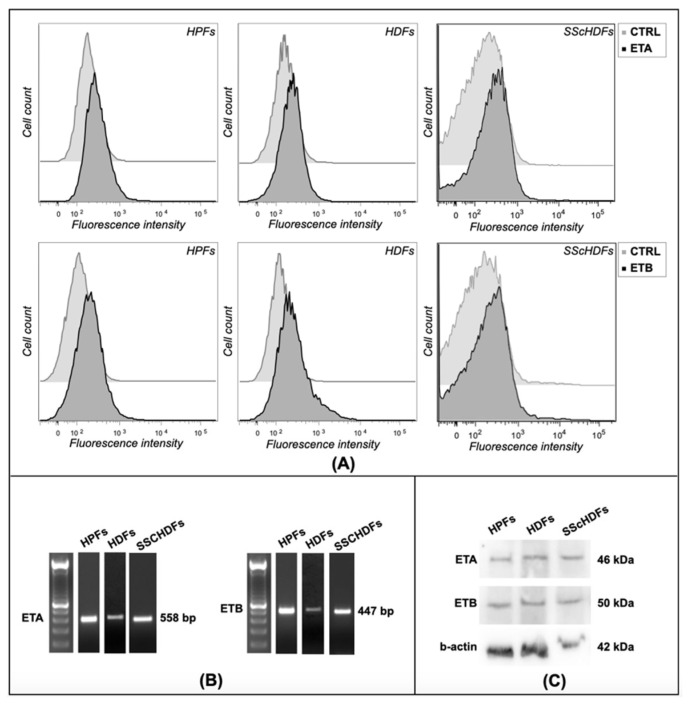
ET receptor detection in fibroblasts. (**A**) Expression of ET receptors was analyzed with flow cytometry, and (**B**) RT-PCR and (**C**) Western blot analysis in HPFs, HDFs, and SScHDFs. Both ET receptors were detected in the cells analyzed both at the mRNA level and at the protein level. (HPFs: human pulmonary fibroblasts; HDFs: human dermal fibroblasts; SScHDFs: scleroderma dermal fibroblasts; ETA: endothelin receptor A; ETB: endothelin receptor B).

The presence of ETA and ETB receptors was assessed in HPFs, HDFs, and SScHDFs: at the transcriptional level evaluating the presence of receptors’ mRNA by RT-PCR and at the protein level by flow cytometry and Western blot analysis. The densitometric analysis of Western blot bands did not show a difference in protein amounts among the cells tested after normalization to β-actin. All of the fibroblasts tested express both ETA and ETB at various levels analyzed (Figure 1).

### 3.2. MCR and CYP11B2

**Figure 2:**
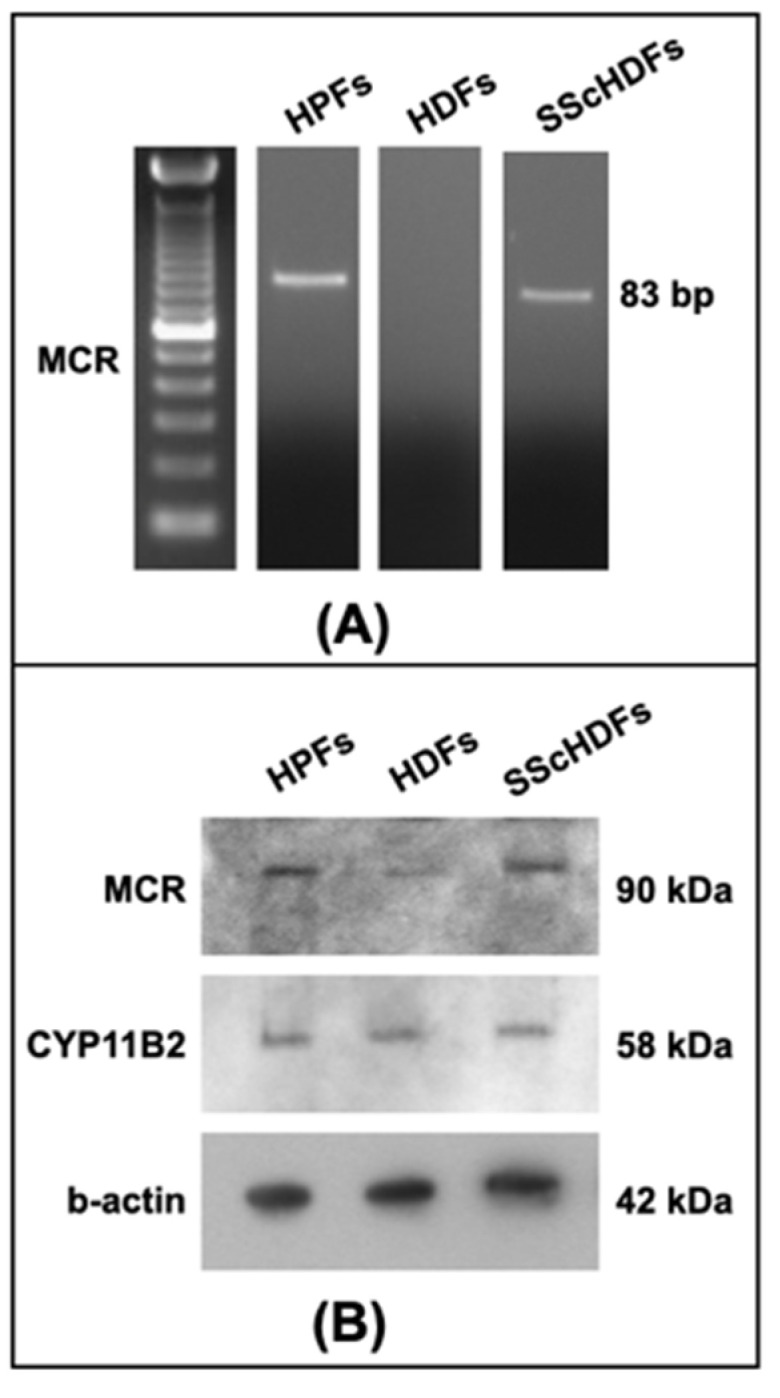
MCR and CYP11B2 expression in fibroblasts. (**A**) RT-PCR showed the presence of MCR mRNA in HPFs and SScHDFs but not in HDFs. (**B**) Western blot analysis confirmed the presence of MCR in HPFs and also in HDFs and SScHDFs. Furthermore, it showed a different receptor amount in cells analyzed: HPFs and SScHDFs expressed more than double the amount of MCR than HDFs (1.27 and 1.03, respectively); the CYP11B2 amount was similar in every kind of fibroblasts. (HPFs: human pulmonary fibroblasts; HDFs: human dermal fibroblasts; SScHDFs: scleroderma dermal fibroblasts; MCR: mineralocorticoid receptor; CYP11B2: cytochrome P450 11B2).

MCR and CYP11B2 were evaluated to better understand if a possible different response of analyzed cells to aldosterone stimulus was due to variations in the receptor or the presence of synthase. Using RT-PCR, HPFs showed the presence of mRNA for MCR, while it seems not to be present in HDFs, but the Western blot analyses of HPF, HDFs, and SScHDFs showed the presence of MCR with a difference in protein amounts among the cells tested after normalization to β-actin: SScHDFs and HPFs express MCR 1.03 and 1.27 times more than HDFs, respectively. The non-detection of MCR in RT-PCR could be due to a small quantity of the mRNA not being amplified by the reverse transcription; in fact, the receptor appears in a low amount at the protein level. The presence of CYP11B2 was evaluated in HPF, HDFs, and SScHDFs by Western blot analysis to better understand the aldosterone biogenesis pathway, and all of these cell types showed the presence of a similar amount of this enzyme (Figure 2).

### 3.3. Trans-Differentiation and Fibroblasts Activation

**Figure 3:**
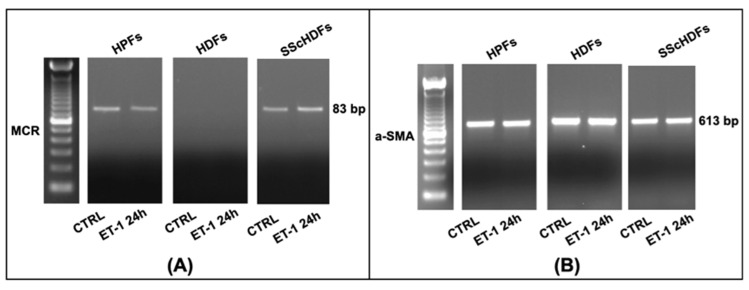
ET-1 effects on MCR (**A**) and α-SMA (**B**) in HPFs, HDFs, and SScHDFs. MCR mRNA was not detectable in HDFs before and after ET-1 stimulation for 24 h, whereas α-SMA mRNA was present before and after ET-1 stimulation for 24 h in HPFs, HDFs, and SScHDFs. (HPFs: human pulmonary fibroblasts; HDFs: human dermal fibroblasts; SScHDFs: scleroderma dermal fibroblasts; MCR: mineralocorticoid receptor; α-SMA: α-smooth muscle actin).

**Figure 4:**
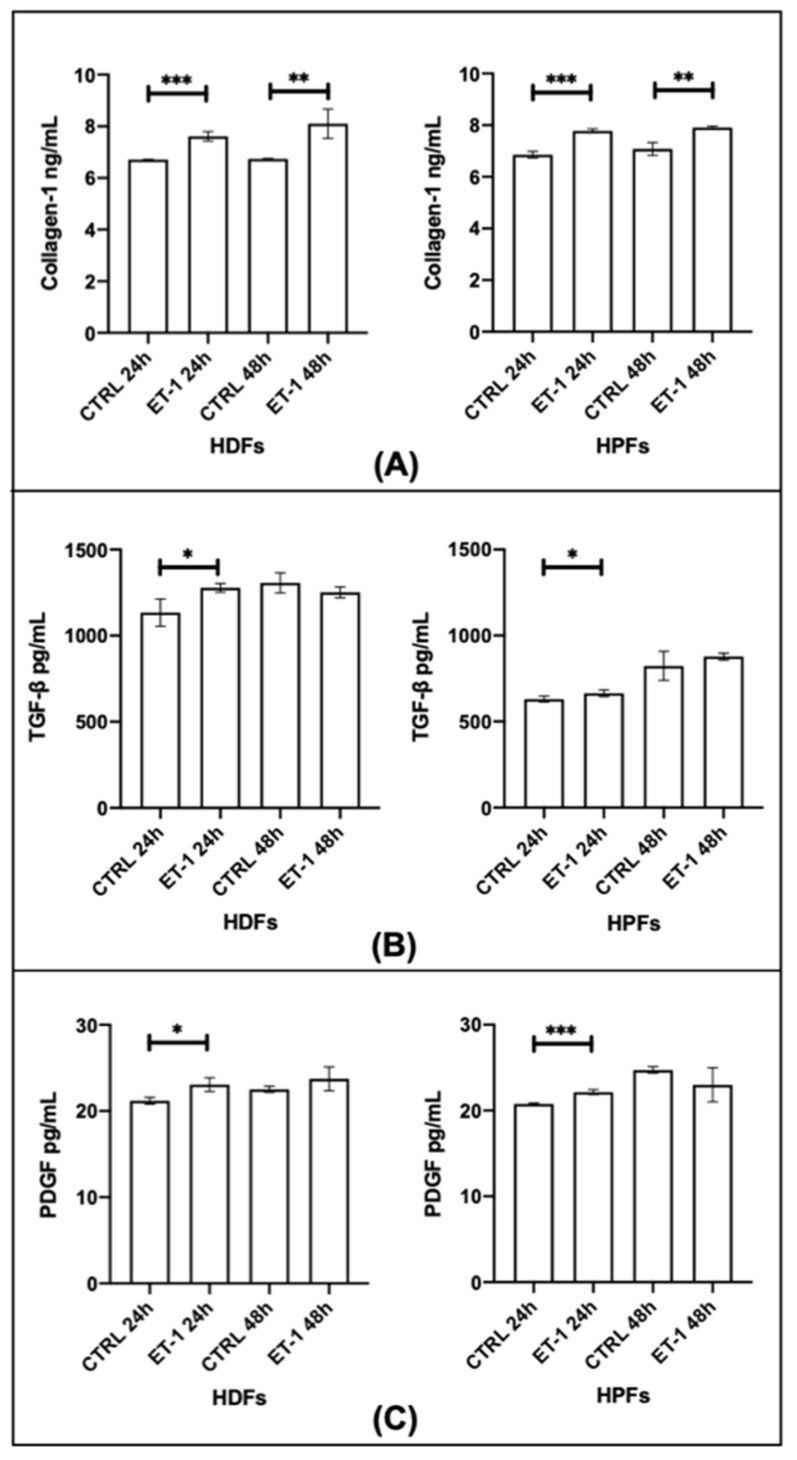
Fibroblast activation after ET-1 stimulation. The activation of fibroblasts was analyzed measuring levels of (**A**) collagen-1 reported in ng/mL, (**B**) TGF-β reported in pg/mL, and (**C**) PDGF reported in pg/mL in supernatants of HPFs and HDFs after 24 or 48 h of incubation with ET-1. A stimulation of 24 h was sufficient to induce the production of collagen-1, TGF-β, and PDGF in HPFs and HDFs. After 48 h, collagen-1 was released by HPF and HDF supernatants. (*** *p* < 0.0005; ** *p* < 0.005; * *p* < 0.05). (HPFs: human pulmonary fibroblasts; HDFs: human dermal fibroblasts; ET-1: endothelin-1; TGF- β: transforming growth factor-β; PDGF: platelet-derived growth factor).

HPFs and HDFs stimulated by ET-1 for 24 h (Figure 3) did not show changes in MCR and α-SMA mRNAs before and after ET-1 stimulation. To the contrary, in HPF and HDF cultures, supernatants, the protein levels of collagen-1, TGF-β, and PDGF before and after ET-1 stimulation for 24 or 48 h changed (Figure 4).

### 3.4. Oxidative Stress

**Figure 5:**
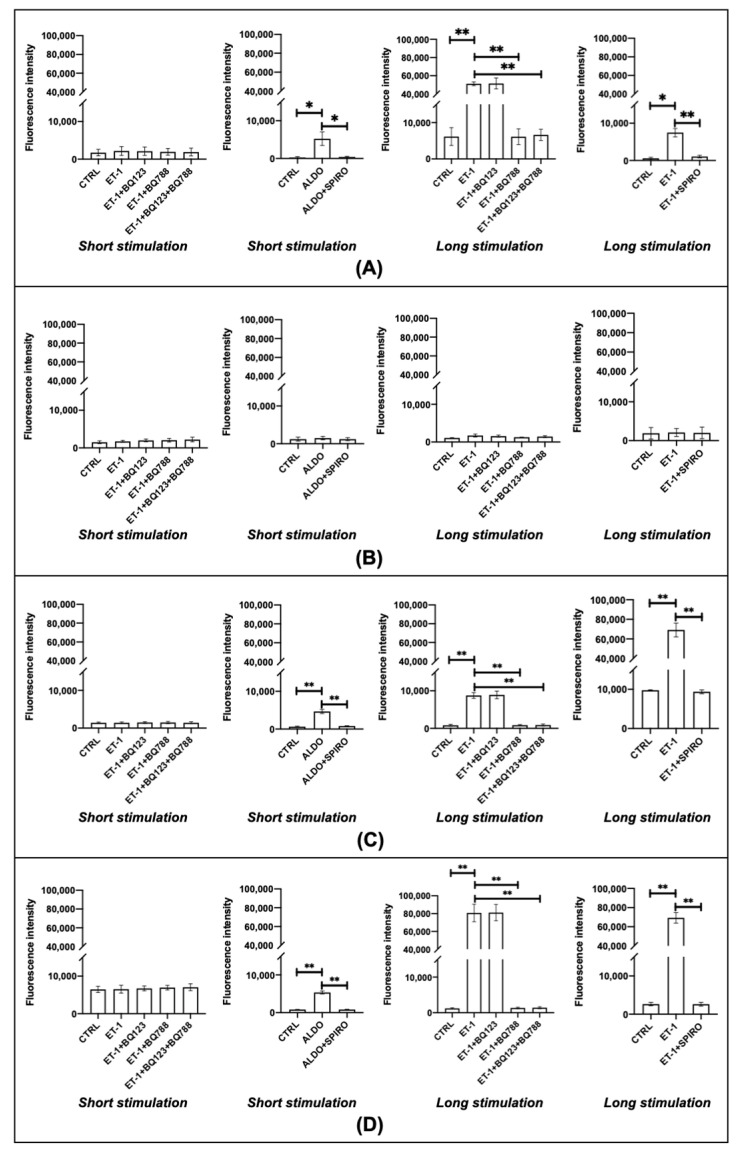
Oxidative stress evaluation in (**A**) HPFs, (**B**) HDFs, (**C**) lSScHDFs, and (**D**) dSScHDFs. All cells analyzed except HDFs produce ROS after a long ET-1 stimulation of 24 h and the antagonist of ETB (BQ788) or aldosterone (spironolactone) inhibits this effect; short aldosterone stimulation of 40 min is sufficient to promote oxidative stress. ROS production was evaluated by measuring the fluorescence intensity of CM-H2DCFDA. (** *p* < 0.005; * *p* < 0.05). (HPFs: human pulmonary fibroblasts; HDFs: human dermal fibroblasts; lSScHDFs: limited scleroderma dermal fibroblasts; dSScHDFs: diffuse scleroderma dermal fibroblasts; ET-1: endothelin-1; BQ123: ETA blocker; BQ788: ETB blocker; SPIRO: spiroronolactone).

**Figure 6:**
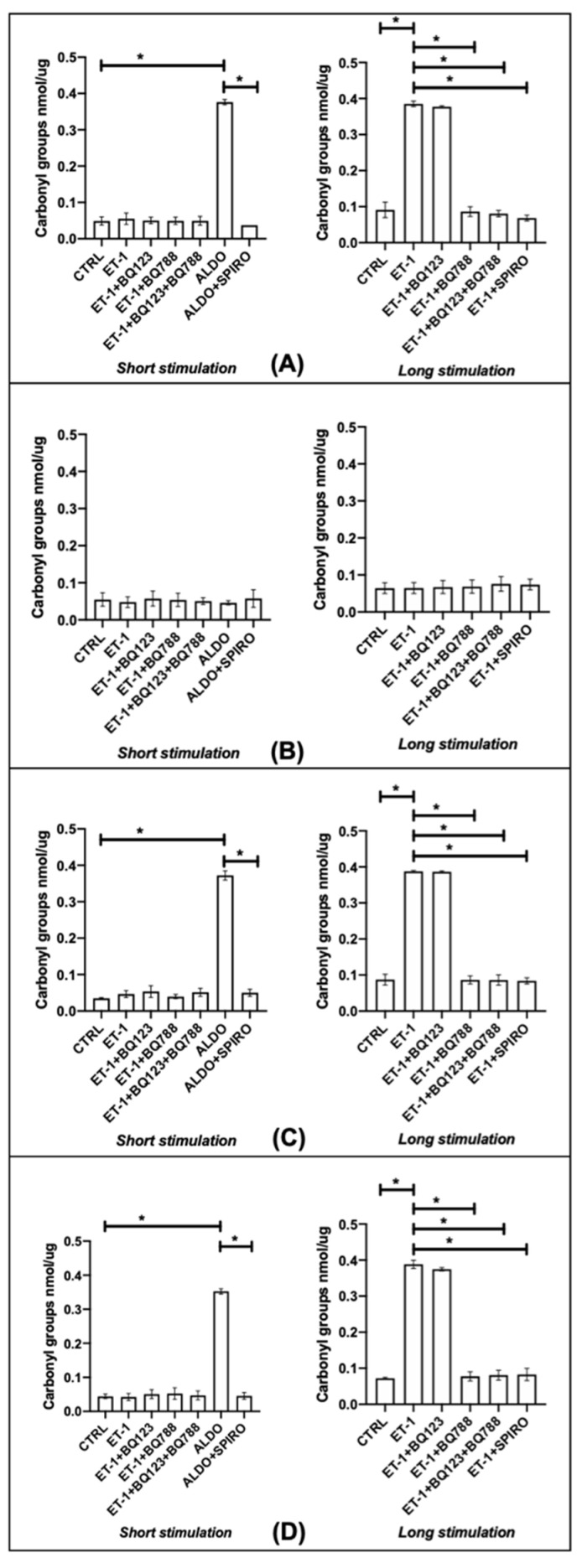
Protein carbonyl content assay in (**A**) HPFs, (**B**) HDFs, (**C**) lSScHDFs, and (**D**) dSScHDFs. In all cells analyzed except HDFs, the amount of carbonyl groups reported in nmol/μg was increased after a long ET-1 stimulation of 24 h and the antagonism of ETB (BQ788) or aldosterone (spironolactone) inhibits this effect; short aldosterone stimulation of 40 min is sufficient to promote oxidative stress. (* *p* < 0.05). (HPFs: human pulmonary fibroblasts; HDFs: human dermal fibroblasts; lSScHDFs: limited scleroderma dermal fibroblasts; dSScHDFs: diffuse scleroderma dermal fibroblasts; ET-1: endothelin-1; BQ123: ETA blocker; BQ788: ETB blocker; ALDO: aldosterone; SPIRO: spiroronolactone).

In HPFs and both limited and diffuse SScHDFs, ET-1 stimulated oxidative stress only after 24 h of incubation but the ETB blocker BQ788 or the combined ETA/ETB blocker inhibited this production; aldosterone was able to stimulate oxidative stress in HPFs and SScHDFs after 40 min of incubation. The behavior of these cells was interesting when stimulated with ET-1 for 24 h and pre-incubated with spironolactone: MCR inhibitor spironolactone was able to inhibit the production of ROS due to ET-1 incubation. In HDFs, there was no ROS production both after 40 min or 24 h with different stimulations. These results were found both measuring the oxidative stress using CM-H2DCFDA (Figure 5) and quantizing the carbonyl groups (Figure 6). With these two different types of analysis, directly by flow cytometry and indirectly by quantification of carbonyl groups, we could be sure about the data obtained.

### 3.5. Aldosterone

**Figure 7:**
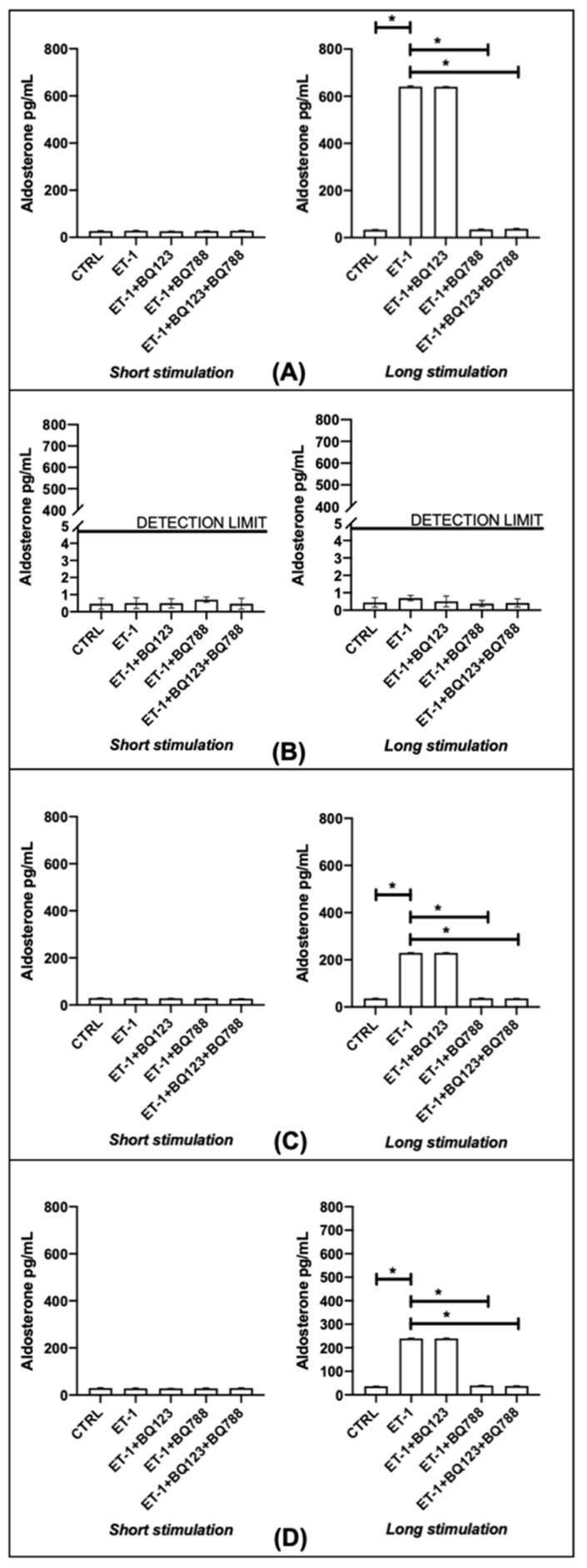
Aldosterone production in (**A**) HPFs, (**B**) HDFs, (**C**) lSScHDFs, and (**D**) dSScHDFs. All cells analyzed except HDFs produce aldosterone reported in pg/mL after long ET-1 stimulation of 24 h, and the antagonism of ETB (BQ788) inhibits this effect. (* *p* < 0.05). (HPFs: human pulmonary fibroblasts; HDFs: human dermal fibroblasts; lSScHDFs: limited scleroderma dermal fibroblasts; dSScHDFs: diffuse scleroderma dermal fibroblasts; ET-1: endothelin-1; BQ123: ETA blocker; BQ788: ETB blocker).

HDFs did not show detectable aldosterone amount (pg/mL) after short or long ET-1 stimulation. HPFs and SScHDFs (both lSScHDF and dSScHDF) showed a statistically significant aldosterone increase only after long incubation but not when the ETB blocker BQ788 or the combined ETA/ETB blocker was used (Figure 7).

## 4. Discussion

ET-1 was firstly identified as a peptide with vasoconstrictive action, but recently its involvement in cell adhesion, angiogenesis, inflammation, and fibrosis has been described. It is worth mentioning that PAH is a severe complication of SSc, in particular of the limited form of the disease; moreover, PAH is the main cause of death in SSc patients [37]. Furthermore, plasma, derma, and internal organs of patients affected by these pathologies show high levels of ET-1 [38,39,40,41]. The ET-1 effects in vitro are not completely understood: on one hand, it stimulates SMCs contraction though ETA, and on the other hand, the stimulation of fibroblasts leads to the production of extracellular matrix proteins [2,24]. The engagement of the ETB expressed on ECs can activate eNOS and increase NO production. This mechanism appears to be in contrast with the ability of ET-1 to stimulate the endothelial synthesis of aldosterone through PPAR coactivator-1α, steroidogenesis factor-1, and CYP11B2, since aldosterone is able to block the ETB signaling pathway. Indeed, a decreased level of NO is found in PAH patients, and, therefore, the treatment approaches consider taking precise advantage of the therapeutic agents mimicking NO action.

With the present study, we investigated the possible role of ET-1 in oxidative stress and in fibroblast activation. Moreover, our aim was to clarify the possible link between ET-1 and aldosterone in inducing oxidative stress. The first step was to confirm the presence of ET-1 receptors ETA and ETB on the fibroblasts used in this study and, in particular, on HPFs, HDFs, and SScHDFs. MCR was found in different amounts in the various cells analyzed, suggesting that fibroblasts’ behavior in different tissues, such as lung and skin, may be different; on the contrary, the amount of CYP11B2 was similar in the different fibroblasts analyzed. The different expression of MCR among HPFs, HDFs, and SScHDF could be one of the causes of skin fibrosis that characterizes patients with SSc, as well as pulmonary fibrosis. The origin or mechanisms underlying these differences deserve further investigation with future studies. EndoMT is a very important feature in the pathogenesis of fibrosing diseases, and for this reason, we decided to evaluate whether a similar process occurred in fibroblasts after stimulation with ET-1. Our results showed that ET-1 did not increase the transcription of α-SMA mRNA, and we therefore hypothesize that cells need a second stimulus to start the trans-differentiation process. Indeed, in the literature, it was reported that activated fibroblasts increased the expression of α-SMA acquiring a contractile phenotype in healing tissues [16]. However, ET-1 was able to increase the production of collagen-1, TGF-β, and PDGF in the supernatant of dermal and lung fibroblasts.

Fibroblasts stimulation with ET-1 for 24 h increased oxidative stress in HPFs that express both ETA and ETB receptors, but only the block of ETB with BQ788 led to a lower production of ROS. On the contrary, the ETA block with BQ123 did not exert any effect on the ROS production. The data obtained in HPFs suggest that the binding of ET-1 to ETB is important for oxidative stress. It is interesting to point out the different behavior of HDFs and SScHDFs, since both kind of fibroblasts express the two ET-1 receptors, but only SScHDFs showed ROS production similar to HPFs. Indeed, HDFs did not produce ROS after the same stimulation, and this difference may be due to the different MCR expression in fibroblasts from different tissues and also from the same tissue in a healthy state and in fibrotic disease.

Since the short incubation with aldosterone was able to induce ROS production in HPFs and SScHDFs, whereas ET-1 needed 24 h of incubation to induce ROS, we hypothesize that aldosterone could be the mediator of the ROS production induced by ET-1. This hypothesis is supported by the absence of ROS production in fibroblasts when they are incubated for 24 h with ET-1 and with the aldosterone inhibitor spironolactone. Moreover, the inhibition of ROS production by ET-1 when incubated with ETB blocker BQ788 but not by incubation with ETA blocker BQ123 suggests that the ET-1/ETB interaction is important for the oxidative stress in both SScHDFs and HPFs. Since the induction of oxidative stress requires an incubation of 24 h with ET-1, it is possible that this incubation time may be necessary for the activation of the signaling cascade for aldosterone synthesis.

**Figure 8:**
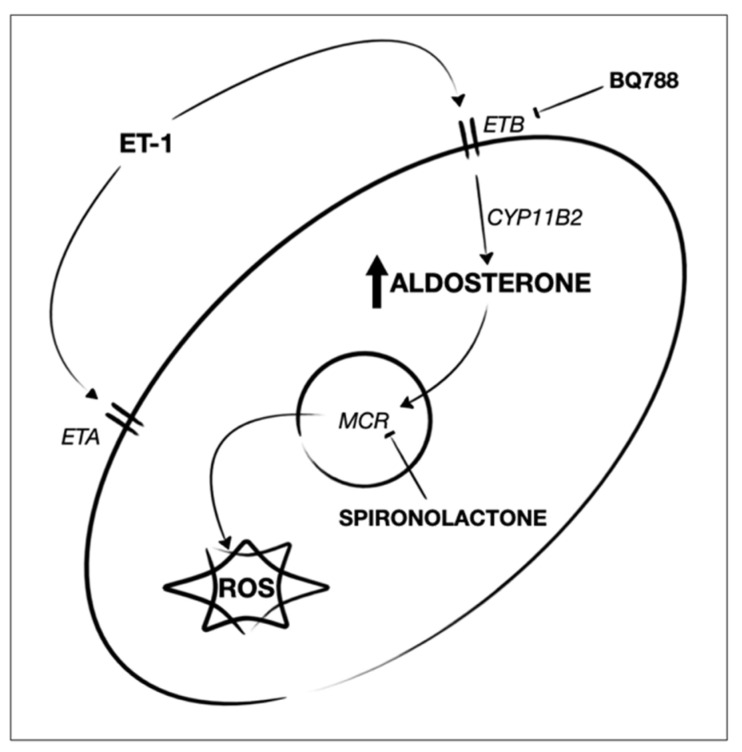
Proposed mechanism. The binding of ET-1 to its ETB receptor activates CYP11B2 increasing aldosterone, which, through its receptor (MCR), induces ROS production in HPFs and SScHDFs. The inhibition of the ETB receptor or the antagonism of MCR arrests this process.

Our results confirm the profibrotic effect of ET-1 by inducing fibrogenic cytokines and collagen-1 secretion by fibroblasts. Moreover, ET-1’s ability to induce ROS production seems to be mediated by the induction of aldosterone that could be truly responsible for oxidative stress, and such effects are mediated by ETB (Figure 8). The data obtained confirm an important role for ET-1 in the pathogenesis of PAH and SSC, reiterating the importance of drugs that act by modulating the endothelin pathway. Endothelin receptor antagonists are already used in the treatment of PAH, associated or not with connective tissue diseases. The impact of mineralocorticoid antagonists in PAH is currently under study, and our data further support the possibility of investigating its efficacy. The use of spironolactone has been shown to be safe and well tolerated in patients with PAH, although data on its efficacy in improving the functional capacity of patients or its effect on the improvement of long-term outcomes are lacking [24,42]. Treatment with another mineralocorticoid receptor antagonist, eplerenone, showed in rats the ability to reduce vascular remodelling and an improvement in right ventricular function, supporting the importance of further studies about the use of mineralocorticoid receptor antagonists [43]. In conclusion, these data suggest that treatment with drugs able to block ETB and inhibit aldosterone may be a rational treatment for SSc and PAH, and therefore they may be considered able to modify the course of disease. Our future perspective is to investigate the effect of clinically available endothelin receptor antagonists, such as bosentan or ambrisentan, on oxidative stress and aldosterone production in fibroblasts; thus, our results may support clinical activity.

## Figures and Tables

**Table 1:** Clinical characteristics of SSc patients and healthy donors.

	Sex	Age	Disease Form	Disease Duration
**Patients**	1 Male 3 Female	34–51	2 dSSc 2 lSSc	3 < 5 years 1 > 5 years
**Healthy donors**	1 Male 1 Female	32–45	-	-

## Data Availability

The raw data supporting the conclusions of this article will be made available by the authors, without undue reservation.
